# Low invasion success of an invasive cyanobacterium in a chlorophyte dominated lake

**DOI:** 10.1038/s41598-019-44737-8

**Published:** 2019-06-05

**Authors:** Sarah Bolius, Claudia Wiedner, Guntram Weithoff

**Affiliations:** 1 Department for Ecology and Ecosystem Modelling, University of Potsdam, Am Neuen Palais 10, 14469 Potsdam, Germany; 2 Leibniz Institute for Baltic Sea Research Warnemünde, Seestraße 15, 18119 Rostock, Germany; 3 Berlin Brandenburg Insitute of Advanced Biodiversity Research (BBIB), Altensteinstr. 34, 14195 Berlin, Germany

**Keywords:** Freshwater ecology, Invasive species, Microbial ecology

## Abstract

Biological invasions are a major threat to biodiversity and ecosystem functioning. Successful invasions depend on the interplay of multiple abiotic and biotic factors, however, the process of the invasion itself is often overlooked. The temporal variation of environmental factors suggests that a ‘window of opportunity’ for successful invasions exists. Especially aquatic habitats, like temperate lakes, undergo pronounced seasonal fluctuations and show temporally varying environmental conditions in e.g. nutrient availability, temperature and the composition of the resident community including competitors and consumers. We experimentally tested if an invasion window for the globally invasive cyanobacterium *Cylindrospermopsis raciborskii* exists. From May to September, we determined the invasion success of *C*. *raciborskii* in laboratory mesocosms with natural lake water. Although the invasion success was generally low, the invasiveness varied among months and differed in total invasive biomass, net development and final share of *C*. *raciborskii* in the community. During the first days, *C*. *raciborskii* strongly declined and this initial, short-term decline was independent of the ambient consumptive pressure. These results are in contrast to laboratory studies in which *C*. *raciborskii* successfully invaded, suggesting that a complex natural system develops a resistance to invasions.

## Introduction

In recent decades, the number of species that spread into new ranges has increased. These invasive species can have several, mostly negative, ecological consequences. Invasive species are common in almost all types of ecosystems, but they are, particularly in aquatic systems an increasing threat to biodiversity and ecosystem functioning^1^. Furthermore, in contrast to larger plants or animals, invasive microbes have attracted much less attention in invasion ecology^2^. One exception is the invasive, sub-tropical freshwater cyanobacterium *Cylindrospermopsis raciborskii* that has successfully invaded temperate lakes^3–5^. The taxonomic position of *C*. *raciborskii* is still under debate, only recently Aguilera *et al*.^6^ suggested to unite the genera *Raphidiopsis* and *Cylindrospermopsis* under the name *Raphidiopsis*. Three general key factors have been identified that drive the invasion success of species^2,7^: (1) the identity and genetic diversity of the invader, (2) the characteristics of the resident community including consumption and competition^8^ and (3) the abiotic characteristics of the habitat. In lakes these are for example nutrient supply^9–12^, temperature^13^ and light availability^14,15^. In the case of *C*. *raciborskii*, all of these three factors have been discussed and their relevance for the invasion success evaluated: (1) The genetic identity and the phenotypic plasticity of *C*. *raciborskii* plays a role in its invasion success, for example, only one out of three tested strains successfully invaded experimental mesocosms^16^. The high degree of phenotypic plasticity facilitates the spread into new geographic regions e.g. through a broad range of pre-adaptations or within-species genetic variation^17,18^. (2) In aquatic systems, the invasion of species is mainly driven by consumptive community resistance^19^, i.e. herbivory/predation is the main factor in reducing the invasiveness of invaders^19^. As a filamentous cyanobacterium, *C*. *raciborskii* is not readily edible^20^ and of low nutritional value as a sole food source for zooplankton^21,22^. However, all classes of zooplankton species can ingest *C*. *raciborskii* and are able to reduce its biomass^21,23–25^ as long as the filament density is low. Furthermore, a reduction of zooplankton favoured the invasion of *C*. *raciborskii* in experimental mesocosms using natural lake water^16^. (3) Two of the main abiotic factors for the invasion of *C*. *raciborskii* are temperature and nutrients^17^. The two main limiting nutrients for autotrophic growth in lakes are phosphorus (P) and nitrogen (N)^26^. However, *C*. *raciborskii* as a member of the Nostocales is diazotrophic i.e. it can fix atmospheric nitrogen by using heterocysts^27–32^. This is an advantageous, but not exclusive trait because other nitrogen fixing species typically occur in temperate lakes. However, *C*. *raciborskii* is rather dominating under P-depleted conditions^33–35^, likely because of its high P-affinity and storage capability^34^. Due to its sub-tropical origin, *C*. *raciborskii* is adapted to high temperatures. For invasive and non-invasive strains, an optimum growth was found at approx. 30 °C^36,37^ much higher than the maximum temperatures in temperate lakes. The minimum temperature for growth is around 11–15 °C^38,39^. An important feature for the population dynamics in temperate lakes of *C*. *raciborskii* is the germination from resting stages (akinetes) after overwintering on the lake bottom^40,41^. The optimal germination temperature is the temperature range between 22 and 24 °C^36,42^. Consequently, the maximum abundance of *C*. *raciborskii* in the temperate zone is observed in summer at temperatures around 22–24 °C e.g.^3,4,38,43,44^. Other abiotic habitat factors associated with a higher abundance of *C*. *raciborskii* are high pH, high conductivity^45,46^ and fluctuating light conditions^38,43,47,48^. In temperate lakes, all these factors regularly fluctuate over the time course of a year^49^ suggesting a seasonal variability in the invasiveness - a ‘window of opportunity’ exists, during which an invasion is more likely than at other time points. This temporal heterogeneity assumes to allow for seasonal invasion niches and promotes coexistence^10,50,51^.

Most knowledge on the invasibility of species and *C*. *raciborskii* was either derived from laboratory studies on their ecology and physiology or from comparative field studies based on observations e.g.^52^. Experimental studies on the invasion success in multi-species laboratory communities or in natural communities and lake water have rarely been performed, though needed to understand invasions in a community context.

In this study, we investigated the invasion success and a potential window of opportunity of the invader *C*. *raciborskii* into a Northeast German lake. We tested the invasive capabilities of *C*. *raciborskii* in a 2 × 2 factorial design under near-natural conditions by using experimental mesocosms with natural water from a lake where *C*. *raciborskii* has not been detected. The two factors were herbivory and the internal phosphorus status. We hypothesize that the invasion success of *C*. *raciborskii* exhibits a clear seasonality with respects to absolute invasion success and temporal variation in the dominant factors. To allow for a high genetic diversity and plasticity, the invading *C*. *raciborskii* population was a mixture of 11 strains originating from Northeast German lakes.

## Methods

We performed invader-addition experiments, for which we have used the ambient abiotic and biotic conditions of the local lake Glindower See (52°21′22.5′′N, 12°56′15.8′′E), near Werder (Havel) in Brandenburg, Germany. *C*. *raciborskii*, the invader, has never been detected in that lake^16^, thus it is a suitable study site for this invasion experiment. The water was collected monthly from May to September 2016 and used to set up mesocosms and *C*. *raciborskii* was added as the invader in laboratory experiments, with different levels of herbivory and manipulated intracellular P-content. The experiments were conducted modified after Weithoff *et al*.^16^.

### Invader

We used 11 different strains of the cyanobacterium *C*. *raciborskii*, isolated from six lakes in Northeast Germany^18^. The strains are genetically different and differ also in their ecophysiological traits, e.g. growth rate, nutrient use, and grazing loss by a generalist herbivore, the rotifer *Brachionus calyciflorus*^18^. All these strains differ slightly in their morphology, but cannot be unambiguously distinguished by microscopy.

### Adapting and cultivation of invading species

To avoid a failed invasion of *C*. *raciborskii* simply by the sudden change of the environment (transfer effect)^16^, all strains were acclimatised to lake water. This was achieved by stepwise (30%, 60% lake water) diluting the cyanobacteria medium (Woods Hole medium, after Nichols^53^; 2 mM HEPES buffer, pH 8, 80 µg phosphorus L^−1^, molar nitrogen: phosphorus ratio = 20:1) with sterile lake water (filtered through 0.2 µm cellulose acetate filters, Sartorius, Göttingen, Germany) up to 100% lake water. Throughout the duration of all experiments the stock cultures were individually kept in sterile lake water. After every monthly sampling this water was substituted and the cultures were kept in sterile lake water of the previous month, which led to varying nutrient ratios due to fluctuating nutrient concentrations in the lake (see Results). This procedure guaranteed stable, continuous growth of all strains; two spot checks in May and June revealed an average daily growth rates of all strains of about 0.25 after the supply of fresh lake water.

### Sampling, experimental design and set up

Each month from May to September, lake water was collected from approx. 1.5 m depth using a 3.5 L Ruttner-type sampler. A zooplankton sample was obtained by filtering 5 L through a 30 µm mesh and fixed with Lugol’s iodine. For phytoplankton, a raw sample was fixed with Lugol’s iodine for subsequent enumeration and species determination. For the analysis of particulate carbon (C) and chlorophyll-*a* (chl-*a*), water was vacuum filtered on glass fibre filters (GF/C, 25 mm; Whatman International Ltd, Maidstone, UK; precombusted for C-analysis). Three fractions of phosphorus were analysed: for soluble reactive phosphorus (SRP), filtrate from a filtration through a 0.45 µm filter (membrane filter, PALL Cooperation, Port Washington, New York, USA) was used; particulate phosphorus (PP) was analysed from that filter and total phosphorus (TP) was analysed from an untreated sample.

**Figure 1:**
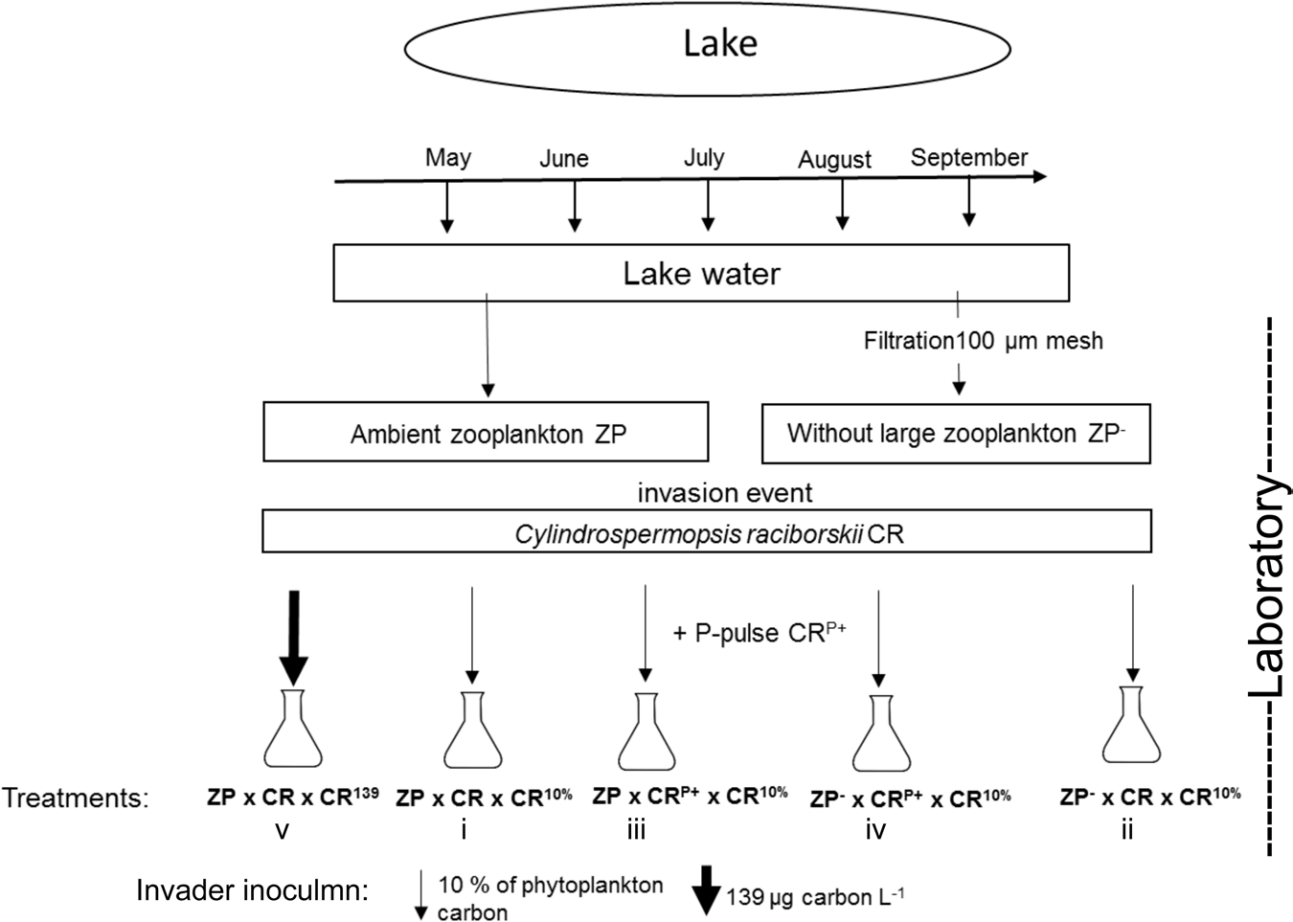
Schematic of the set-up for the monthly experiments. Treatments: ZP = ambient zooplankton density, ZP^−^ = without large zooplankton, CR = *Cylindrospermopsis raciborskii* with normal cellular phosphorus(P)-content, CR^P+^ = *C*. *raciborskii* with increased cellular phosphorus-content, CR^10%^ = addition of 10% *C*. *raciborskii* (in µg carbon, arrows) of the lake phytoplankton community, CR^139^ = addition of a fixed amount of 139 µg carbon L^−1^ *C*. *raciborskii* (bold arrow).

Five different treatments with the addition of *C*. *raciborskii* (CR) were set-up (Fig. 1) using a 2 × 2 factorial design with manipulations of the zooplankton density and the internal P-content of *C*. *raciborskii*: (i) ambient zooplankton (ZP) density (ZP × CR × CR^10%^), (ii) without large zooplankton (ZP^−^ × CR × CR^10%^), (iii) ambient zooplankton with increased cellular P-content of *C*. *raciborskii* (ZP × CR^P+^ × CR^10%^), (iv) without large zooplankton and with increased cellular P-content (ZP^−^ × CR^P+^ × CR^10%^). The additions of *C*. *raciborskii* to the treatments (i–iv) amounted for 10% (in µg C) of the lake phytoplankton community. As a fifth treatment (v) a fixed amount of *C*. *raciborskii* of 139 µg C L^−1^ was added to a treatment with ambient zooplankton density and unenriched P-content (ZP × CR × CR^139^), which was ca. 1.5 to 5 times higher than in the other treatments depending on the sampling date (Table 1). The increased intracellular P-content of the filaments (CR^P+^) shall mimic the physiological state of filaments shortly after germination from akinetes, which are supposedly rich in nutrients. All treatments were set up in quadruplicate, 20 mesocosms each month.

**Table 1:** The monthly inoculum and carbon to phosphorus (C:P) ratio of *Cylindrospermopsis raciborskii*, which was added to the lake community. CR is *C*. *raciborskii* without and CR^P+^ with a P-pulse overnight and subsequent lower C:P ratio.

Cylindrospermopsis raciborskii					
	May	June	July	August	September
Inoculum carbon µg L^−1^ CR^10%^	35	55	96	61	27
C:P CR	266:1	146:1	176:1	117:1	282:1
C:P CR^P+^	93:1	44:1	50:1	54:1	177:1

At the sampling day, 800 ml of lake water was filled into 1 L Erlenmeyer flasks. For the zooplankton removal, water was sieved through 100 µm mesh, which has been proven to remove large species while keeping the phytoplankton abundance and composition unaffected^16^.

The day before the invasion event, aliquots of the 11 *C*. *raciborskii* strains were mixed after measuring the optical density (OD) and diluted to equal amounts in terms of carbon using pre-established conversion factors (OD at 800 nm, UV Mini 1240 UV-VIS spectrophotometer, Shimadzu, Kyoto, Japan). From that mixture a subsample was filtered on glass fibre filter for carbon analysis (see below). For increasing the intra-cellular P-content, half of the mixture was spiked with phosphate overnight. The concentration of this P-pulse was calculated from the carbon content of *C*. *raciborskii* and its P-uptake per unit carbon^18^. The phosphate addition was chosen to allow for substantial P-uptake and P-reduction in the medium to avoid significant P-input into the mesocosms. On average, the P-pulse increased the intra-cellular P-content by a factor of ca. 3, resulting in a corresponding decreased C:P ratio (CR^P+^, Table 1).

The following day, the filters for particulate carbon determination of the lake water and *C*. *raciborskii* were analysed (see below) and from these values the inoculum of *C*. *raciborskii* was calculated (Table 1) and added to the mesocosms as the invasion event. Temperature and light:dark cycle in the climate chamber were adjusted to the ambient environmental conditions (see Table 2). The light intensity was the same during all months (approx. 130 photons µm s^−1^ m^−1^). Every experiment lasted for 24 days, every third day 20% (160 ml) of the volume were substituted with sterile lake water, which was obtained from the same sampling occasion. At the end of the experiments, water was filtered for carbon, nitrogen, chlorophyll-*a*, PP and SRP analyses (as above).

**Table 2:** Lake parameters of the sampling months, obtained at 1.5 m. C = carbon, chl-*a* = chlorophyll-*a*, N = nitrogen, P = phosphorus, part. = particular, cellular, SRP = soluble reactive phosphorus.

Lake parameters					
	May	June	July	August	September
temperature °C	16.8	23.5	24	21.8	20.2
pH	7.9	8.5	8.5	8.2	7.9
Secchi depth m	4.8	2.5	2.25	3.25	2.75
part. C:N:P mol	77:15:1	81:21:1	115:23:1	55:19:1	51:14:1
total P µg L^−1^	99	68	114	142	188
SRP µg L^−1^	76	40	68	95	153
chl-*a* µg L^−1^	5	10	11	14	5
Light:dark h	15:9	16:8	16:8	15:9	12:12

### Analyses

For each of the five experiments, the initial abundance and composition of the resident phytoplankton and zooplankton was analysed. For phytoplankton, the standard Utermöhl method was applied using an inverted microscope (AxioObserver, Carl Zeiss, Jena, Germany), individual cells were measured (AxioVision Zen 2, Carl Zeiss) and the cell volume was calculated assuming suitable geometric shapes^54^. The phytoplankton carbon content was calculated from the biovolume, following the equation of Rocha and Duncan^55^. Zooplankton was enumerated and the carbon content calculated according to established conversion factors: for rotifers by Telesh *et al*.^56^; for *Bosmina*, copepods and *Daphnia* spp. it was assumed that 40% of the dry weight is carbon^57^ and the dry weight was calculated following the correlation between dry weight and body length^58^. The abundance of *C*. *raciborskii* was determined at day 3 and 24 to distinguish between short-term survival and long-term establishment.

The carbon and nitrogen values were analysed using an elementary analyser (EA 3000, EuroVector S.p.A., Milan, Italy). The phosphorus was determined photometrically following the molybdate blue method of Murphy and Riley^59^ at 880 nm (UV Mini 1240 UV-VIS spectrophotometer, Shimadzu). For the particulate and total phosphorus, cells were digested with H_2_SO_4_ and K_2_S_2_O_8_ and 1 h autoclaving at 121 °C prior to the analysis. Chl-*a* was extracted in 60 °C hot 90% ethanol overnight, measured fluorometrically^60^ using pre-calibrated chl-*a* standards (TD 700, Turner Designs, Sunnyvale, California, USA).

For statistical analysis of the differences between months and treatments either an ANOVA (when data were normally distributed) or Kruskal-Wallis test (for non-normal distributed data) was performed. The correlation between the success of *C*. *raciborskii* and factors was analysed by linear regression with SPSS (25, IBM) and by a principal component analysis (PCA) in R (R Development Core Team, 2010; RStudio 1.0.136, package ‘stats’). At the end of the experiments, we calculated the relative share of *C*. *raciborskii* in the total phytoplankton community as the calculated *C*. *raciborskii* carbon content (assuming a conversion of pg C = 0.15 * biovolume [µm^3^]) divided by total particulate carbon and the net development i.e. the relative change in biomass over time corrected for the regular dilution – as a measurement for its invasion success. When *C*. *raciborskii* was not detected (in some mesocosms), we used half of the lowest biomass of the invader as a zero-replacement value.

## Results

We conducted invasion experiments with *C*. *raciborskii* to identify its potential ‘window of opportunity’ to invade a lake in Northeast Germany using 11 strains isolated from this region.

### Abiotic factors and initial resident compositions of the lake

Glindower See is a eutrophic lake with high SRP and TP concentrations throughout the investigation period (Table 2).

The temperature increased from 16.8 C° to its maximum in July of 24 °C and decreased until September to 20.1 °C. Along the increase in temperature, water transparency declined from 4.8 m to 2.25 m and the chl-*a* concentration increased.

**Figure 2:**
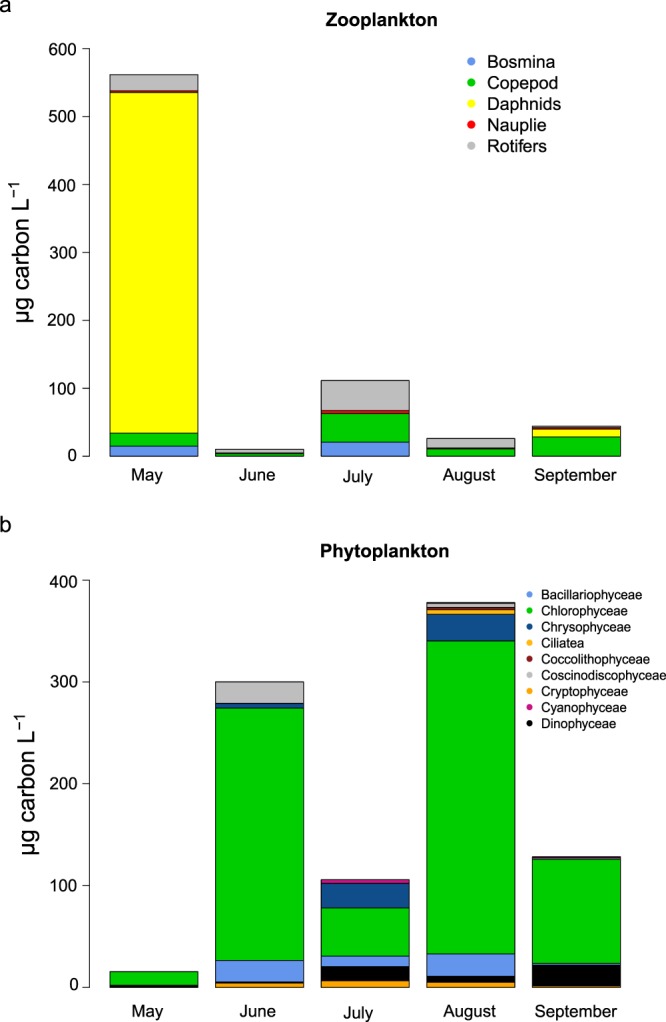
Composition of (**a**) zooplankton and (**b**) phytoplankton communities at the sampling day of each month.

Phyto- and zooplankton abundance and composition varied strongly over the season (Fig. 2): in May, *Daphnia* spp. species dominated, and phytoplankton abundance was low. The opposite relation was found in June and August: lower zooplankton biomass (10, 25 C µg L^−1^), was associated with higher phytoplankton biomass. The phytoplankton community consisted mainly of *Chlamydomona*s sp. and other Chlorophyta and Bacillariophyceae species, among the zooplankton, *Pompholyx sulcata*, copepods and *Keratella cochlearis* were most abundant taxa. During July, there was a slight increase of zooplankton: *Bosmina*, more copepods and rotifer species were abundant, which also resulted in a decrease in the total phytoplankton abundance. The main rotifer species were *K*. *cochlearis*, *P*. *sulcata* and *Synchaeta stylata*.

### Nutrients at the end of the experiments

After the 24 day lasting experiments, the available SRP was highest in September with 75 µg P L^−1^ (Table 3) and much lower in the other month ranging from 8 to 24 µg P L^−1^ (Kruskal-Wallis test H = 68.79, *p* < 0.001; Dunn’s Post-hoc *p* < 0.005). At the end of the experiments chl-*a* was highest in July with an average of 42 µg L^−1^, in August and September it was around half of it with 26 µg L^−1^ and 15 respectively 16 µg L^−1^ in May and June. The particulate carbon content was similar in May, July and September with on average around 4 mg L^−1^ and lower in June and August with an average of 2.5 mg L^−1^. This difference between months is significant between May and August and September and between August and September (Kruskal-Wallis test, H = 20.65, *p* < 0.001; Dunn’s Post-hoc *p* < 0.05).

**Table 3:** The average nutrient concentrations and ratios of the community at the end of the experiments (day 24). C = carbon, chl-*a* = chlorophyll-*a*, N = nitrogen, P = phosphorus, SRP = soluble reactive phosphorus.

Lake parameters (day 24)					
	May	June	July	August	September
C:P mol	187:1	266:1	303:1	85:1	128:1
N:P mol	17:1	47:1	43:1	10:1	12:1
C:N mol	12:1	6:1	7:1	9:1	11:1
SRP µg L^−1^	20	8	12	24	76
chl-*a* µg L^−1^	15	16	42	26	26

The C:N ratio was highest in May with on average 12:1 and significantly different compared to these from June to August (ANOVA, F = 18.92, *p* < 0.001; Tukey Post-hoc *p* < 0.01). The C:P ratio was highest in July with an average ratio of 303:1 with significant differences to the low ratios in August and September (85:1, 128:1; Dunn’s Post-hoc *p* < 0.002). At the end of the experiments, in most months, intracellular P-content decreased and C:P ratio increased.

### Invasion success of *Cylindrospermopsis raciborskii*

**Figure 3:**
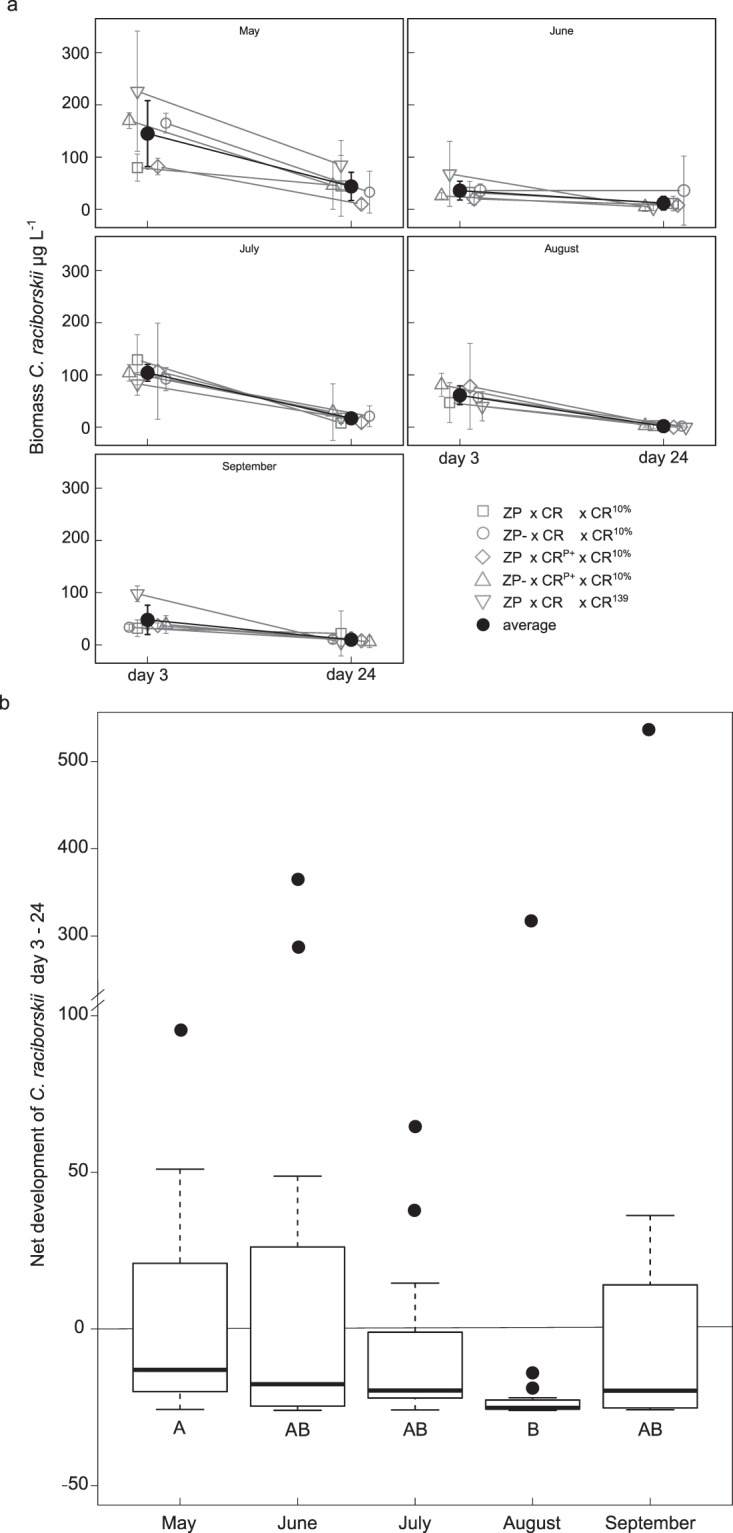
Invasion success of *Cylindrospermopsis raciborskii* in the monthly experiments. (**a**) The biomass of *C*. *raciborskii* (µg L^−1^) at day 3 and 24. The single treatments are plotted in grey with open symbols, the average of the months is plotted as a full black circle. Treatments: ZP = ambient zooplankton density, ZP^−^ = without large zooplankton, CR = *C*. *raciborskii* with normal cellular phosphorus-content, CR^P+^ = *C*. *raciborskii* with increased cellular phosphorus-content, CR^10%^ = addition of 10% *C*. *raciborskii* (in µg carbon) of the lake phytoplankton community, CR^139^ = addition of fixed amount of 139 µg carbon L^−1^ *C*. *raciborskii*. Mean ± SD, N = 20. (**b**) The net development of *C*. *raciborskii* between day 3 and 24 from May to September. It is the observed loss of *C*. *raciborskii* in relation to the expected remaining biomass of 26% due to dilution. A positive value implies a positive net development. Letters indicate the homologous subgroups of the Dunn’s Post-hoc test (Kruskal-Wallis test, H = 14.92, *p* = 0.005; Dunn’s Post-hoc *p* = 0.002). Bold line = median, dots are outliers, N = 20.

In all monthly experiments, *C*. *raciborskii* declined strongly within the first days and maintained only low abundances until the end of the experiments (Fig. 3a). A drastic decline of biomass was observed within the first three days of the experiments, when *C*. *raciborskii* lost on average 520 µg L^−1^ (83%). The highest decrease was in August with a loss of 93% of its biomass.

The highest abundance, after three days, of *C*. *raciborskii* has remained in May with a biomass of 145 µg L^−1^, followed by July with on average 104 µg L^−1^. The abundance of *C*. *raciborskii* in May and July was significantly different from the other months. At day 24, the biomass in May significantly differed from all other months, except July, the biomass in July was only significantly different to August (day 3: Kruskal-Wallis test, H = 53.638, *p* < 0.001; Dunn’s Post-hoc test *p* < 0.022; day 24: Kruskal-Wallis test, H = 15.707, *p* = 0.003; Dunn’s Post-hoc test *p* < 0.032). Between day 3 and 24 the biomass loss was continuously high of around 79% (62 µg L^−1^). This decline of *C*. *raciborskii* led to a very low remaining biomass at day 24, with either a biomass between 2 (August) and 44 (May) µg L^−1^ (Fig. 3a) or no detectable filaments of *C*. *raciborskii*, detected in 30% (30) of the mesocosms. This decline roughly equalled the experimental dilution.

Assuming no growth of *C*. *raciborskii* during the experiments, the dilution rate of 20% every three days would have led to a decline of 74% biomass from day 3 until day 24. In most flasks, the abundance of *C*. *raciborskii* was below that level or *C*. *raciborskii* disappeared, in a few others, the final abundance was higher (positive value > 26%, negative value < 26%, Fig. 3b). Positive values, indicating a positive net development, were found in 37% of the mesocosms. In four single flasks, the net development was very high (outliers, Fig. 3b, in June, August and September). A significant difference was found between May and August (Kruskal-Wallis test, H = 14.92, *p* = 0.005; Dunn’s Post-hoc *p* = 0.002).

**Figure 4:**
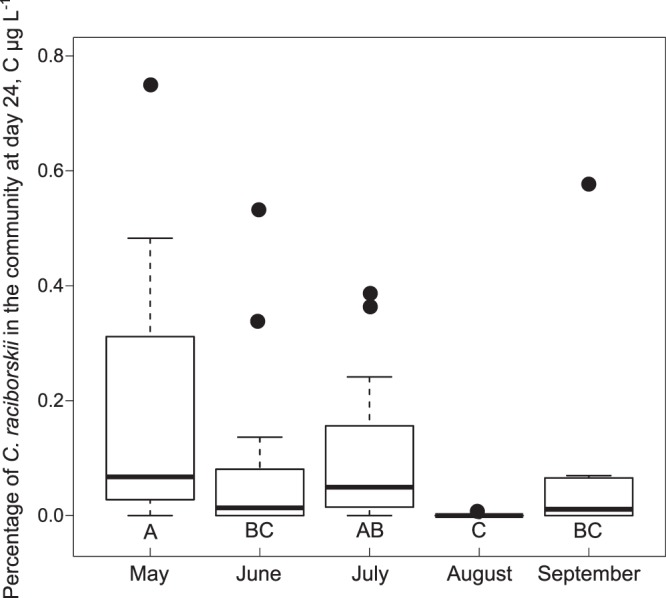
Percentage of *Cylindrospermopsis raciborskii* of the total particulate carbon at day 24 of the monthly experiments plotted as boxplots. The total particulate carbon is a measurement for the phytoplankton abundance. Letters indicate the homologous subgroups of the Dunn’s Post-hoc test (Kruskal-Wallis test, H = 32.965, *p* < 0.001; Dunn’s Post-hoc *p* < 0.036). Bold line = median, dots are outliers, N = 20.

As an alternative measure for the invasion success, we calculated the share of *C*. *raciborskii* to the total phytoplankton community measured as the particulate carbon at day 24 (Fig. 4). This ratio is significantly different among months (Kruskal-Wallis test, H = 32.965, *p* < 0.001; Dunn’s Post-hoc *p* < 0.036), however, the share of *C*. *raciborskii* in the remaining community was continuously low and did not exceed 1%.

Overall, the different treatments had no or only minor effects on the invasion of *C*. *raciborskii*. Only in May, the lower zooplankton abundance and the higher propagule pressure of *C*. *raciborskii* (treatments ii, iv and v) led to a slightly higher invader abundance after 3 days (Dunnett’s Post-hoc test *p* < 0.018) compared to the treatments with ambient zooplankton. In September, the higher propagule pressure (v) had a positive effect on the invasion, leading to a nearly 3-fold higher abundance after 3 days (ANOVA, F = 18.45, *p* < 0.001). Towards the end of the experiments these differences levelled off.

3 days after invasion, the biomass of *C*. *raciborskii* was positively correlated to Secchi depth (linear regression, R^2^ = 0.295, *p* = 0.005) and negatively to temperature (linear regression, R^2^ = 0.206, *p* = 0.023). The other environmental factors of the lake (C, chl-*a*, light:dark cycle, pH, PP, TP) were not significantly correlated to the biomass of *C*. *raciborskii* (day 3: R^2^ > 0.009, *p* > 0.225). The *C*. *raciborskii* abundance at day 24 was likewise correlated to temperature (linear regression, R^2^ = 0.275, *p* = 0.007) and Secchi depth (linear regression, R^2^ = 0.307, *p* = 0.004), suggesting a seasonal effect.

**Figure 5:**
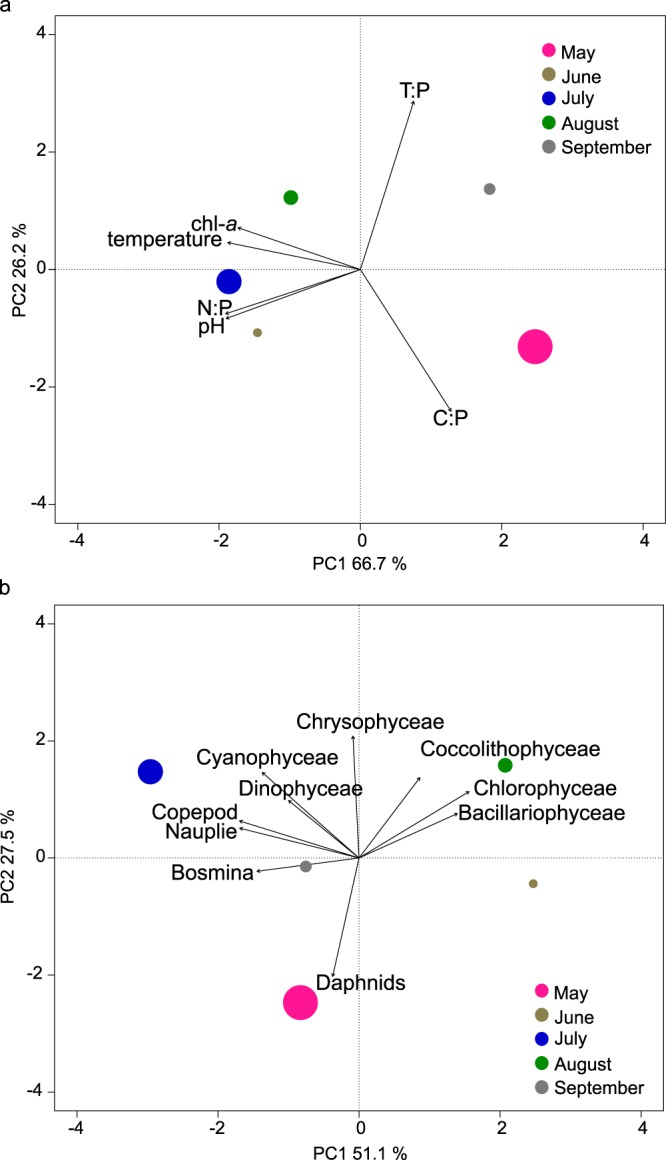
Principal component analysis ordination of the experimental set-ups based on (**a**) the abiotic factors and (**b**) the species composition of the sampling day. The size of the points represents the monthly abundance (biomass µg L^−1^) of *Cylindrospermopsis raciborskii* at day 3.

These seasonal differences were also visible in the PCA. The principal component analysis revealed a strong explanatory power (93% of the first two axes, Fig. 5a) of the physical and chemical environmental factors, mainly by pH, chl-*a* and the N:P ratio. An ordination based on the abundance and composition of zooplankton and phytoplankton (Fig. 5b) showed a lower though still quite high explanatory power: the different zooplankton and phytoplankton groups mainly drove the ordination along axis 1. Daphnids and Crysophyceae explained the ordination along axis 2.

## Discussion

The success of invasive species is, besides the characteristics of the invader itself, determined by the habitat and its species composition. Since the environmental characteristics in temperate lakes change considerably over time, we hypothesised a clear seasonal pattern in the invasion success of *C*. *raciborskii*. Overall, a seasonal effect on the invasion success of *C*. *raciborskii* was detectable, but on a very low level (Figs 3, 4), despite strong seasonal differences in species composition and environmental factors (Figs 2, 5 and Tables 1, 2). The months with the most opposing environmental conditions, May and August (Fig. 5a, Table 2) differed most in the abundance and relative share of *C*. *raciborskii*. Especially within the first three days, *C*. *raciborskii* lost most of its biomass, resulting in the low invasion success. The invader could only maintain a low amount of biomass until the end of the experiments. This suggests no ideal ‘window of opportunity’ to invade the Glindower See. Based on the relative biomass, *C*. *raciborskii* persisted best in May and July, two months with almost opposing abiotic factors and different community compositions.

The relatively moderate invasion success of *C*. *raciborskii* in May and July, expressed as a comparable high biomass ratio, was associated with the lowest phytoplankton abundance, which kind of ‘opened’ the invasion window for *C*. *raciborskii* (Figs 2, 3, 5b). The low phytoplankton abundance in May and July was likely because of zooplankton grazing (Fig. 2). In May, the zooplankton community was mainly dominated by *Daphnia* spp., who are able to ingest filaments and suppress *C*. *raciborskii* blooms only when filament densities are low^61^. For this month, the removal of large zooplankton led to a lower decline of *C*. *raciborskii* within the first three days, suggesting a top-down effect. According to results from a similar study, the environment at the time point of invasion is of crucial importance for the invasion success; over the time course of the experiment, differences might level off due to the regular dilution of the water^16^. In July, the community consisted mainly of small rotifers (40%, mainly *K*. *cochlearis*) and copepods (38%) and no effect of the zooplankton removal was found. Copepods are more specialists and selective grazers^62^ and their impact on *C*. *raciborskii* is supposedly low^63^, thus their removal had only very limited effects^64^. Likewise, the smaller rotifer *K*. *cochlearis* is often associated with the presence of *C*. *raciborskii* in lakes^65^ and might feed less on filamentous species. Moreover, in July, the abundance of other cyanobacteria in the community was higher than in the other months (Figs 2b, 5b) suggesting suitable conditions for cyanobacterial growth. Although *C*. *raciborskii* was added proportionately to the ambient phytoplankton communities, a high natural phytoplankton abundance might hamper the invasion through a high collective competitive strength. Thus, the relative share of *C*. *raciborskii* was only low compared to that of the other species (Fig. 4). Nevertheless, the slightly higher invasion success and share of *C*. *raciborskii* in May and July coincided with high intracellular C:P ratios and low SRP concentrations at the end of the experiments, underlining the high competitive strength of *C*. *raciborskii*. The low share in August and September was related to higher phosphorus concentrations and lower C:P ratios i.e. conditions when competition was of minor importance. The effects of the different treatments (Fig. 1) on the invasion were also low. This suggests that the environment or the experimental conditions were disadvantageous for *C*. *raciborskii*. Overall, the environmental factor combinations in Glindower See appear to have favoured chlorophytes over cyanobacteria during the whole season. These abiotic conditions with a high phosphorus content and high P:chl-*a* ratio are not uncommon for lakes in Northeast Germany that are connected to rivers^66^. These lakes are often dominated by chlorophytes, cryptophytes or diatoms and cyanobacteria occur only intermittently. Unfortunately, we have no data on the nitrogen concentration in the lake which would allow us to discuss the relation between invasion success and nutrient condition in more detail. In a study from Danish lakes^67^, it was found that in shallow nutrient-rich lakes, chlorophytes were favoured over cyanobacteria by a combination of continuous nutrient inputs and high phosphorus levels.

In a similar study at the same lake^16^, three different strains were tested separately for their invasiveness under different levels of herbivory. In that experiment, two out of three strains completely failed to invade the system and one strain maintained a population at a low level, when the herbivory was reduced. None of those strains was acclimatised to the lake water prior to the experiments. In the present study, *C*. *raciborskii* had been acclimatised to the lake water four weeks prior to the experimental addition, but this procedure did not facilitate the invasion. It appears that the strong decline within the first three days overruled both, the seasonal and the treatment effect.

Another aspect for the population dynamics is the germination of akinetes. Since in our cultures *C*. *raciborskii* never produced akinetes, this factor can be ruled out in the experiments. For the development in the lake the situation might be different. But since *C*. *raciborskii* has never been observed in Glindower See (although it might have been overlooked during routine sampling regimes), the potential for a substantial “akinete bank” on the sediment is low, if present at all. The temperature window for germination in the lake would be during the summer months from June to August.

For *C*. *raciborskii*, most laboratory studies on its ecology and its invasion potential were performed in laboratory monocultures or with communities of very few species with only one trophic level. Alternatively, comparative studies were performed analysing data sets from many lakes within a region (environmental and presence/absence data) to extract a general pattern for the causes of *C*. *raciborskii* abundance and distribution e.g.^45,68^. Ecological studies focussing on the actual invasion process have been rarely conducted. For example, laboratory studies demonstrated that *C*. *raciborskii* can invade ‘artificial’ communities with herbivory^23^ and without^35^. The present study aimed for a near-natural experimental approach within a community context. However, in this more complex community context, the invasion of *C*. *raciborskii* was very low or rather failed. In a comparable study^69^, six different algal species (diatoms, green algae and cyanobacteria) were tested for their invasion potential in natural pond communities and in all cases the invasions failed. None of those species was acclimatised to the pond water prior to the experiment. Considering the acclimatisation, our results suggest that a sudden change into a new and complex community and environment strongly reduces the invasion success. We can only speculate about the reasons for the large discrepancy between the near-natural and purely laboratory experimental outcomes. However, the (micro-)biological community continuously produces chemical compounds such as secondary metabolites that might affect the performance of invading i.e. not adapted species. Allelopathy is suggested to be one further factor driving invasions^70^ and is associated with *C*. *raciborskii*^71^ and assumes a higher competitive resistance.

A common notion is that microbes disperse easily on a very broad scale. Taking this into account, one may assume that different genotypes of microbial invaders enter a new habitat simultaneously. Theoretical considerations suggest that a higher genetic diversity enhances the invasion success of populations^72^. We accounted for that genetic invader diversity by introducing 11 genotypes in equal abundance to all lake communities. Since all of them were isolated from the same geographical area as the study lake, they were potentially adapted to the climatic conditions. For example, all strains grow at 20 °C and at similar phosphorus-conditions in laboratory experiments^18^. Assuming that individual strains responded differently in the experiments i.e. some declined stronger than others, some strains might have been successful invaders. Since the strains cannot be unambiguously differentiated by microscopy, we measured the average invasion success allowing for a ‘sampling effect’, a higher diversity increases the chances that one or more pre-adapted strains were introduced^73^. If only one strain was able to grow under the ambient conditions, then this strain would have needed more time to reach the initial total biomass of all strains. The time between an invasion event and the spread or population increase of the species is called ‘lag time’ and can vary substantially among species and habitats^74^. Often, species are overlooked before they establish a certain population density^75^. This ‘detection threshold’ depends on the size of the species and the habitat and especially in aquatic systems on the sampling frequency^76^. During the first days, *C*. *raciborskii* lost most of its biomass, even though they were acclimatised to the lake water to avoid a transfer effect^16^. The time course of the experiments might have been too short for *C*. *raciborskii* to adapt to the lake conditions and to build up a higher population density^77^. Thus, it might be that, at the end of the experiments, *C*. *raciborskii* was still in its lag phase. However, in all months, in some treatments outliers occurred, which had a higher share and net development (Figs 3b, 4). This points to some undetected or stochastic factor.

We tried to mimic the ambient conditions from the lake in our mesocosms as good as possible (temperature and light:dark rhythm), but we could not simulate turbulence or mixing intensity. However, the results from our experiments concur with the absence of *C*. *raciborskii* and the low contribution of other cyanobacteria to the lake phytoplankton. Taking into account that the invader was acclimatized to the experimental conditions, our results suggest that more complex biological factors hampered the invasion success.

In conclusion, we did not find specific conditions, which are (most) favourable for an invasion of the aquatic cyanobacterium *C*. *raciborskii* under the given abiotic and biotic conditions. During the initial phase of the invasion process, it decreased strongly and, in most cases, maintained on a very low level. In aquatic systems, consumptive resistance is often higher than the competitive resistance^19^, a mechanism which was apparent only in May, when a reduction of zooplankton promoted the invasion success. We could also show an effect of the competitive resistance and after the initial decline, the further establishment is dependent on in part environmental conditions like nutrient supply and temperature. A successful invasion appears to be related to the interplay of several factors on a temporal scale. Even though the abiotic and biotic conditions were favourable for *C*. *raciborskii* we did not observe a successful invasion. These results are diverging from laboratory invasion experiments, and further comparative (semi-) field studies are lacking. This ‘negative’ example shows that more experiments under natural conditions are necessary to understand and predict the invasion of species. Thus, we suggest to conduct more experiments using natural lake water and to disentangle short- and long-term invasion success and establishment of species.

## Data Availability

The datasets generated and analysed during the current study are available from the corresponding author on reasonable request.
